# Nanomedicine in the Topical Management of Vulvovaginal Candidiasis: An Overview of In Vivo Data

**DOI:** 10.3390/jof12080562

**Published:** 2026-08-01

**Authors:** Adriana Lima, José das Neves

**Affiliations:** 1 i3S—Instituto de Investigação e Inovação em Saúde, Universidade do Porto, Rua Alfredo Allen 208, 4200-135 Porto, Portugal; up202005274@edu.fe.up.pt; 2 ICBAS—Instituto de Ciências Biomédicas Abel Salazar, Universidade do Porto, Rua de Jorge Viterbo Ferreira 228, 4050-313 Porto, Portugal; 3 FEUP—Faculdade de Engenharia, Universidade do Porto, Rua Dr. Roberto Frias, 4200-465 Porto, Portugal; 4 IUCS—Instituto Universitário de Ciências da Saúde, CESPU, Rua Central de Gandra 1317, 4585-116 Gandra, Portugal

**Keywords:** azoles, animal models, nanoparticles, nanotechnology, polyenes, women’s health

## Abstract

The continuous increase in vulvovaginal candidiasis cases worldwide is a global health concern that requires being addressed using innovative approaches. The current incidence of recurrent cases of the disease is particularly alarming, and this is not predicted to decrease in the coming years. Virulence factors of *Candida* spp. are still not entirely understood and the relevance of resistance to antifungal treatment, as well as differences between *Candida* species, must be considered when designing therapies. In this review, the key concepts of vulvovaginal candidiasis are introduced, and the potential of nanotechnology-based approaches being developed for mitigating the infection is highlighted. Several nanosystems proposed in recent years (including drug-loaded nanoparticles and intrinsically antifungal nanomaterials) are presented and their efficacy, as tested in animal models, is discussed in detail. Limitations of in vivo models are also specified and considerations for future work are addressed.

## 1. Introduction

Vulvovaginal candidiasis (VVC) is one of the most frequent types of vaginitis, affecting roughly 70–75% of women at least once in their lifetime [1,2]. Despite typically featuring only mild genital symptoms and being easily managed with antifungal therapy, the infection can relapse and even lead to recurrence (RVVC) [3]. These last cases are characterized by the onset of four or more episodes within a period of twelve months, presenting moderate to severe genitourinary symptomatology. It is estimated that RVVC affects 138 million women yearly [4,5], and this number may rise by 15% until the end of the decade [6]. Such numbers highlight the relevance of VVC a pervasive health concern, particularly given their detrimental consequences to the mental health and overall quality-of-life of affected women. There is still much to understand concerning the emergence and development of VVC and its recurrent form, which often seem to be idiopathic [7,8]. Current antifungal therapy is well-established, although mostly stagnant for the last few decades, with only a few truly relevant drug products being introduced in clinical practice [9,10]. Thus, further research needs to be conducted to enable a more efficient and permanent resolution of the disease.

One interesting approach to developing new therapies involves the use of nanotechnology principles and tools to formulate innovative medicines [11]. These nanomedicines appear particularly appealing for the local management of infection, being able to provide interesting features such as improved vaginal distribution and residence, reduced toxicity, protection of drug payloads and control of their release, or enhanced drug–fungi interactions [12,13,14,15]. Information on engineering, production, physicochemical characterization and general in vitro biological behavior aspects of such nanosystems has been extensively described and can be found elsewhere [16,17,18]. Therefore, this review discusses the therapeutic potential of nanotechnology-based approaches currently being developed for the promotion of successful antifungal therapy against VVC. In particular, we focus on nanosystems that have been tested in vivo using animal models.

## 2. Key Concepts in Vulvovaginal Candidiasis Therapy

**Figure 1:**
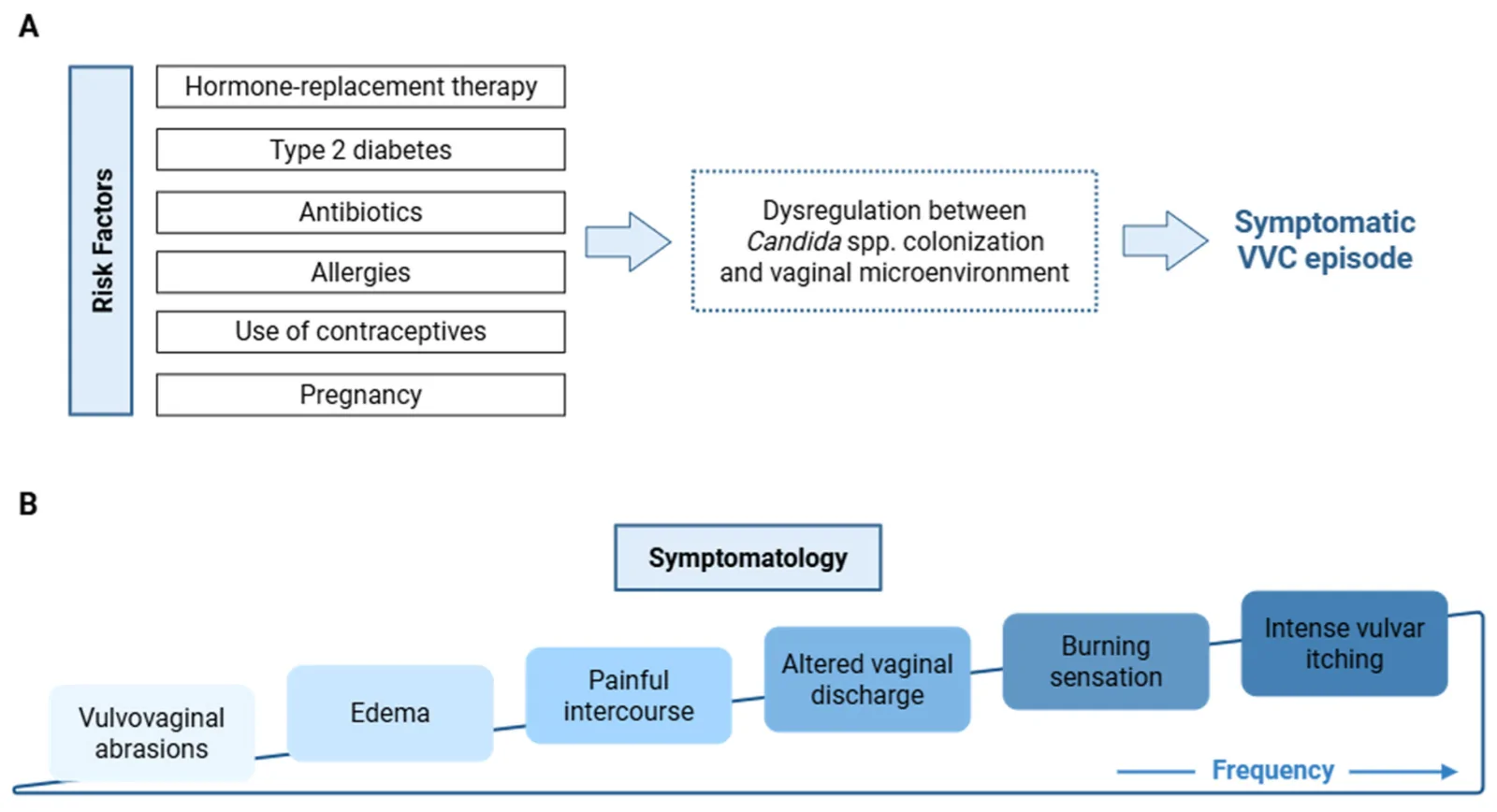
Onset and severity of VVC. (**A**) Schematic representation of main risk factors for the development of VVC and (**B**) common symptoms by frequency of occurrence.

VVC is an infection of the lower female genital tract caused by species of *Candida*. These fungi are part of the normal microbiota in human mucosal surfaces, including the vaginal cavity [19]. Imbalance between *Candida* spp. and the host can promote the development of VVC [20,21]. Infection starts with fungal adhesion to the vaginal epithelium and yeast-to-hyphae (dimorphic) transition, resulting in superficial tissue invasion [22]. Although the disease is regarded as multifactorial and its onset is not fully understood, various factors have been demonstrated to contribute to the occurrence of infection (Figure 1A) [1,23]. *C. albicans* is the most common species responsible for VVC, causing up to about 90% of cases [24]. These infections are usually characterized by mild to moderate symptomatology (Figure 1B) and are easily managed with available antifungals. Cases where non-*albicans Candida* (NAC) species are involved tend to be more severe and complicated to treat, often requiring long-course therapy [25]. Prolonged disease contributes to psychological and mental health effects and reduced quality of life and may result in considerable burden on the overall health of affected women [26,27].

Typical drug therapy for uncomplicated VVC involves the use of azoles by the oral (e.g., fluconazole) or the vaginal route, with a high success rate in uncomplicated infections [28,29]. Conversely, cases of RVVC tend to relapse after cessation of maintenance treatment with azoles, which is also the standard approach for complicated recurrent infections [30]. Azole resistance is also an emerging threat that requires the use of alternative drugs [31,32,33]. NAC species, in particular, are more (or even intrinsically) resistant to azoles [34]. Still, alternative local treatments using boric acid, nystatin, flucytosine or amphotericin B are often subpar, thus justifying the development of new therapeutic approaches. Indeed, the introduction of truly innovative medicines for managing VVC has been meager over the past decades, with the exception of ibrexafungerp, an oral triterpenoid approved in 2021 by the US Food and Drug Administration [35,36].

## 3. Potential of Nanotechnology-Based Approaches in Vulvovaginal Candidiasis

Topical VVC therapy has long been recognized as providing a safe and effective alternative to oral antifungals [37]. That alone justifies why antifungal nanosystems have mainly found application to VVC when considering localized treatment. In particular, the complex characteristics of the cervicovaginal cavity pose considerable obstacles to drug delivery and the use of antifungal nanocarriers can be an interesting way to overcome them. Cervicovaginal mucous fluids present a mesh-like structure formed by interactions between the mucins and other elements of the mucosal layer and act as a stringent barrier to the transport of many drugs and particulates [38,39]. Adequate engineering of size and surface properties of nanocarriers can, however, allow near diffusive transport and contribute to enhanced cervicovaginal distribution and retention of incorporated drugs [40]. Indeed, one of the main issues with nanosystems administered in the vagina is their inability to reside locally for an extended period of time, as the natural cleansing mechanisms of the vagina oppose this retention [41]. Early approaches attempted to enhance the mucoadhesive behavior of nanosystems. However, this also leads to their inability to spread widely throughout the vaginal cavity and limits the ability to reach the underlying epithelial layer, thus inhibiting drug delivery and penetration at the tissue level [42]. Conversely, mucus-penetrating nanosystems can better promote distribution and contact with the epithelial lining, although interactions with epithelial cells may be impaired to a certain extent [43]. These effects lead to enhanced vaginal retention of nanosystems and associated drugs, and even more extensive tissue permeation and absorption [44,45]. In the specific case of vaginal infections, both approaches seem valid and have been adopted for VVC management. Still, the effects of the substantial changes induced to cervicovaginal mucous fluids by the onset of infection on the performance of nanosystems have not been studied and further work is required [46,47].

**Figure 2:**
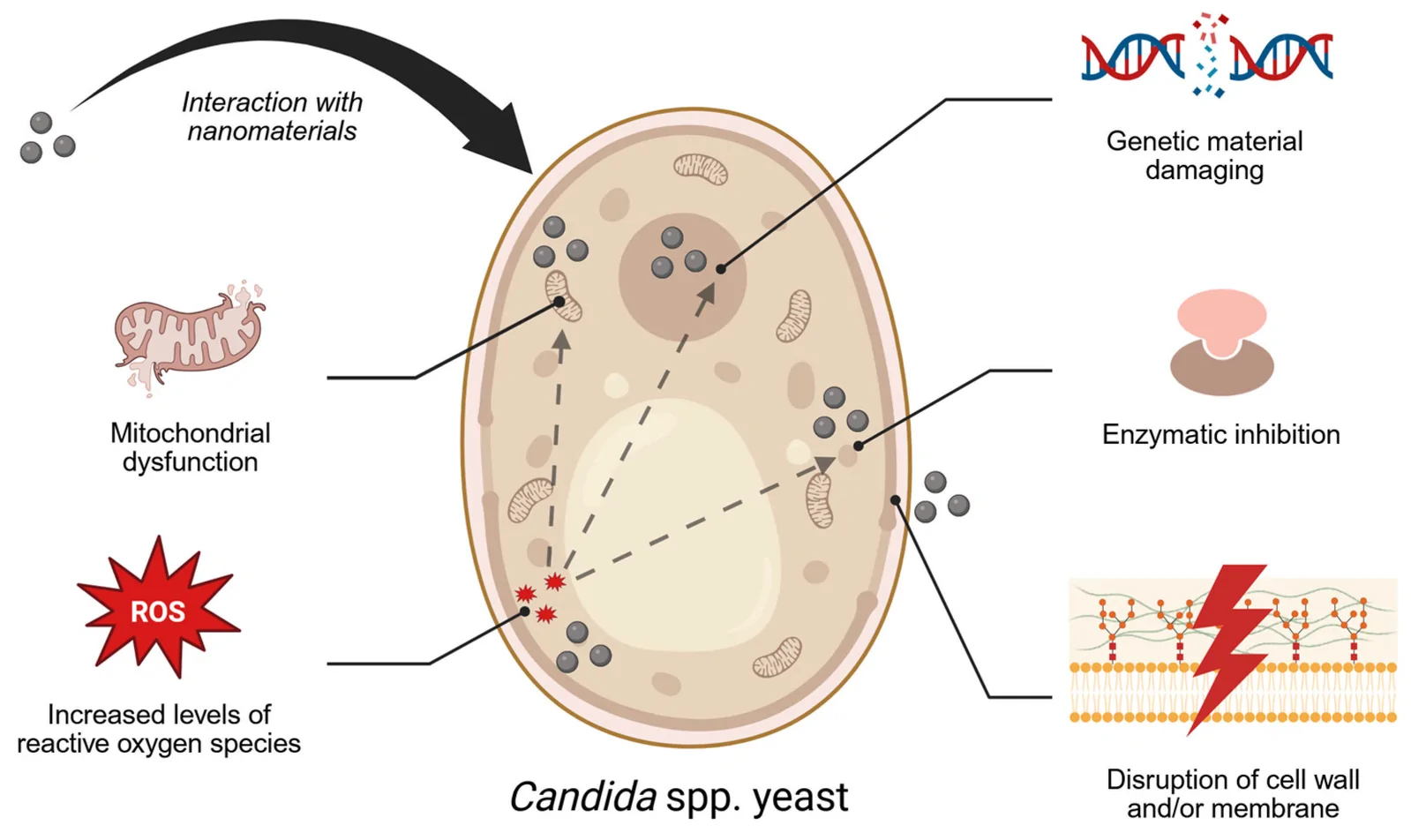
Common mechanism of intrinsic anti-*Candida* activity of nanomaterials. Damage to the fungus can occur solely at the cell surface (cell and/or membrane) or upon internalization and direct interaction of nanomaterials with intracellular components or after release of their components (e.g., metal ions). Dashed arrows indicate secondary action of ROS.

Beyond considerations pertaining to generic challenges posed by the vaginal environment, nanosystems can also be engineered to directly interact with *Candida* pathogens, promoting focal drug targeting or fungal inhibition due to intrinsic antimicrobial activity [15,48]. In this last case, the ability to cause disruptive interfacial interactions at the cell wall and/or membrane [49,50,51], increase the levels of reactive oxygen species (ROS) [52,53], interfere with genetic material synthesis [54], inhibit enzymatic activity [49] or promote mitochondrial dysfunction [54] are common mechanisms leading to antifungal activity (Figure 2). This too has been an area of minute study, but one that likely could propel the utility of nanomedicines to another level. *Candida* spp. is also able to establish biofilms in VVC, and tackling the barrier provided by the fungal extracellular matrix can be challenging [55]. Specific strategies for engineering biofilm-penetrating nanosystems are scarce, but general cues from mucus-diffusive nanoparticles (NPs) [56] or actively transported nanomotors [57] could be helpful in the future.

## 4. In Vivo Efficacy of Nanomedicines Against Vulvovaginal Candidiasis

The general use of nanotechnology-based approaches for combating a variety of *Candida* spp. infections has been widely explored and revised over multiple recent publications [58,59,60,61]. However—and even when intended for specific application to VVC—most of these studies were only conducted at the in vitro level, with no demonstration of in vivo efficacy and safety. Therefore, this section solely focuses on the analysis of published studies testing antifungal nanosystems (either acting as drug delivery systems or used for their intrinsic antimicrobial activity) for managing vaginal infection by *Candida* spp. in relevant animal models of candidiasis. Different studies have been aggregated by incorporated compounds, except for the case of nanomaterials with intrinsic antifungal activity. A summary of discussed studies is presented in Table 1.

### 4.1. Azoles

Azoles (oral triazoles or topical imidazoles) are the first-line choice for clinical management of VVC and, thus, an obvious choice for association with nanocarriers. In particular, topical imidazoles present issues such as the possible onset of local reactions (e.g., irritation, pruritus, burning sensation) [75] and poor solubility that may be potentially circumvented by formulation nanotechnology-based approaches.

A few azole-based nanosystems have been tested in animal models of vaginitis by *Candida* spp., leading to interesting results. For example, Amaral et al. [62] investigated the therapeutic effect of miconazole-loaded chitosan NPs (diameter around 200 nm and zeta potential of approximately +30 mV) using a pseudoestrus-induced BALB/c mouse model challenged with *C. albicans* ATCC 10231. Chitosan NPs were selected due to their well-known mucoadhesive properties and the possibility to sustain drug release. The efficacy of daily intravaginal administration (20 μL) over a 7-day course of miconazole nitrate was evaluated by sacrificing the animals on the day following the last treatment and assessing fungal burden. Results showed no difference between mice treated with miconazole nitrate-loaded NPs at a low dose and animals treated with a miconazole nitrate cream formulation. Histopathological analysis of hematoxylin-eosin-stained sections of vaginal tissue was also performed, denoting no differences between treatments. Both miconazole nitrate-based formulations led to a reduction in the number of hyphae associated with the epithelium and a decrease in immune cell infiltration, suggesting a reduction in tissue inflammation. However, the concentrations (and doses) of miconazole nitrate reported to have been administered in both cases were substantially higher than those used in clinical practice, which could partially limit the relevance of the results.

In a more recent study, the same group explored the synergistic effect of fluconazole in combination with propolis (a natural resinous substance rich in phenolic compounds produced by bees), which was co-associated with similar chitosan NPs (317 nm, around +37 mV) [63]. The in vivo antifungal efficacy of this formulation was determined using the same mouse model and by assessing fungal burden and histopathological features of the vaginal tissue following once daily intravaginal treatment over the course of seven days (20 µL containing 22 mg/kg of body weight for propolis and 2.4 mg/kg of body weight for fluconazole). Researchers observed a 69% reduction in the recovered fungal burden when drug-loaded NPs were used, as compared to animals treated only with phosphate-buffered saline (PBS). This outcome was similar to the 71% reduction in fungal burden of animals treated with a commercially available 2% miconazole cream. Histological analysis showed no signs of inflammation after treatment with NPs, as opposed to what was observed for the group treated with PBS. These results suggest that fluconazole/propolis-loaded chitosan NPs may be a useful option for developing new medicines for managing VVC, although the potential benefits of a synergistic effect between both compounds were not demonstrated due to lack of testing of additional controls (for example, chitosan NPs incorporating only propolis or only fluconazole).

Another imidazole (miconazole) was utilized in a study conducted by Teixeira et al. [64], in which the efficacy of hyaluronic acid-coated, drug-loaded NPs was assessed in a murine model of *Candida* vaginitis. The NPs (211 nm, −53 mV) were administered to female Wistar rats in an induced pseudoestrus phase, with a concentration of miconazole corresponding to 0.004 mg/kg. The treatment was performed two days after the inoculation with *C. albicans* ATCC 10231 with a single intravaginal administration. Hyaluronic acid-coated, miconazole-loaded NPs were able to fully eradicate the pathogen in five animals at 24 h post-treatment, while non-coated counterparts and miconazole in solution were only partially effective (four out of five animals). Regarding the histological analysis, the vaginal tissue of animals that received the hyaluronic acid-coated, miconazole-loaded NPs demonstrated the absence of any histopathological alterations, thus suggesting the safety of the treatment.

### 4.2. Polyenes

A few polyenes, particularly amphotericin B and nystatin, are commonly used as antifungals in clinical practice. Their high affinity to interact with fungal ergosterol and change the permeability of the fungal cell membrane is responsible for activity [76]. Amphotericin B is a broad-spectrum drug that has demonstrated potent therapeutic effects against different yeasts, including *Candida* spp. [77,78]. Its use in clinical practice is typically reserved for the treatment of life-threatening infections, being used by the intravenous route. However, antifungal therapy with amphotericin B is associated with considerable safety issues due to its short therapeutic window and associated nephrotoxicity [79]. Some of these problems have been reduced with different liposomal formulations, with Ambisome being the most notorious one [80,81]. Still, amphotericin B has been proposed as an off-label option for topical management of azole-resistant VVC, but its formulation is challenging due to low aqueous solubility [82,83]. Thus, nanocarriers have emerged as a valid approach for the vaginal delivery of amphotericin B.

**Figure 3:**
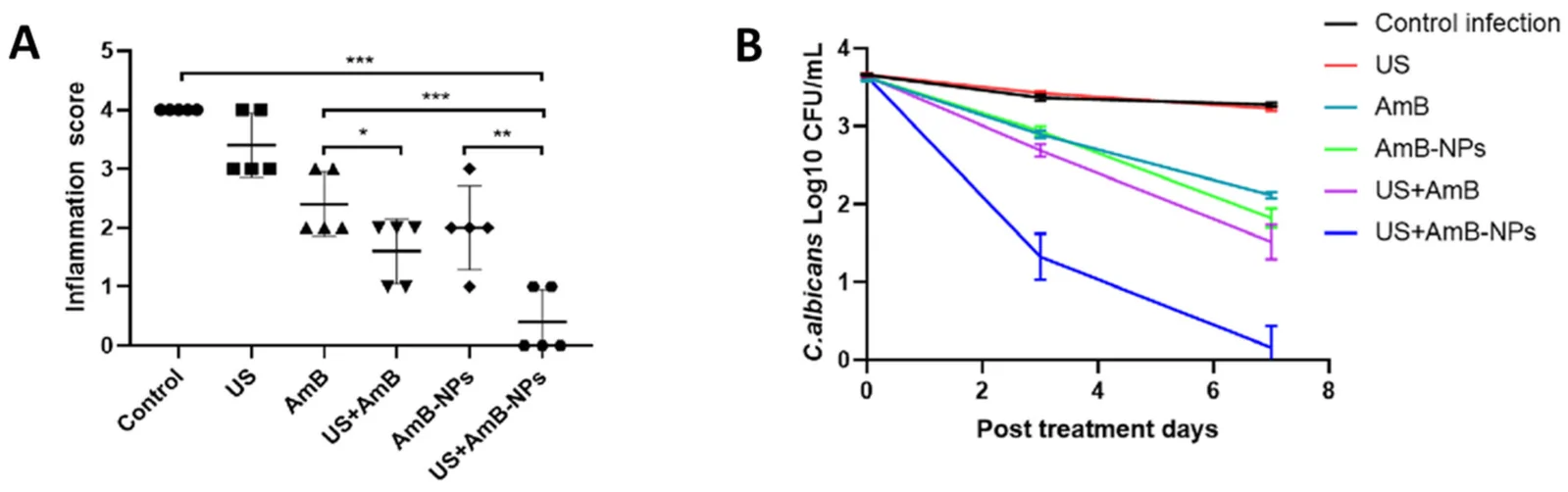
Evaluation of antifungal efficacy of amphotericin B-loaded NPs in a rabbit model of *Candida* vulvovaginitis. (**A**) Vulvovaginal inflammation score calculated for different intravaginal treatment groups based on the vulva hyperemia, redness and swelling, mucosal ulceration and white secretion. (**B**) Quantitative analysis of fungal colony counts (Log10 CFU/mL) of the vaginal lavage fluid in animals infected with *C. albicans* on the zero, third, and seventh day post-treatment. Data represent mean ± standard deviation of initial vaginal fungal burden (*n* = 5 per group). *** *p* < 0.001, ** *p* < 0.01, * *p* < 0.05. Control: intravaginal sterile saline solution; US: application of intravaginal ultrasound; AmB: intravaginal free amphotericin B; US + AmB: intravaginal free amphotericin B followed by application of US; AmB-NPs: intravaginal amphotericin B-loaded NPs; US + AmB-NPs: intravaginal amphotericin B-loaded NPs followed by application of US. Modified from [65], under the terms of the Creative Commons Attribution 1.0 International License (Copyright 2023, Yang et al., https://doi.org/10.1186/s12951-023-01800-x).

For example, Yang et al. [65] proposed poly(lactic-*co*-glycolic) acid (PLGA) NPs with a polyethylene glycol (PEG) coating as delivery vehicles (252 nm, −22 mV) for amphotericin B. These NPs showed lower toxicity to macrophages and lactobacilli when compared with free amphotericin B and were safe when administered intravaginally to healthy female New Zealand rabbits (5 mg/kg/day for 3 days), denoting no signs of nephrotoxicity. The antifungal activity provided by the nanoformulation was evaluated in vivo in immunosuppressed, pseudoestrus-induced New Zealand rabbits infected intravaginally with *C. albicans* ATCC 10231. Animals were treated intravaginally with NPs (corresponding to 0.2 mg of drug) once daily for 3 days, with or without the local irradiation of adjuvant low-intensity ultrasound. The application of ultrasound was intended to increase mucosal permeability and control drug release, and the combination was key for effective treatment of infection (Figure 3). Still, translation of this approach to clinical practice may be hampered by practical and safety issues related to the use of ultrasound [84]. Additionally, the selected rabbit infectious model was not well characterized, namely when compared to more commonly used murine models, thus hampering the interpretation of obtained results.

Another amphotericin B-loaded nanoformulation aiming to improve the treatment of RVVC was developed by Melo et al. [66]. The particles in question were composed of an acrylic/methacrylic acid ester copolymer (Eudragit RL 100) core coated with hyaluronic acid. This last polymer was included to promote attachment to vaginal epithelial cells and promote intracellular uptake. The nanosystem (148 nm, −30 mV) was able to sustain drug release and reduce initial burst, while featuring the capacity to inhibit *C. albicans* growth in vitro. The in vivo efficacy was evaluated in immunosuppressed (pre-treatment with cyclophosphamide), pseudoestrus-induced Wistar rats challenged with *C. albicans* ATCC 14053 and receiving a single intravaginal treatment of 100 µL of the formulation (dose of amphotericin B in the system ranged from 16.2 to 18.3 µg across batches). Evaluation of CFU values was performed in vaginal lavages collected at 24 h after treatment. Results showed the elimination of *C. albicans* from the vagina in animals treated with the hyaluronic acid-coated, drug-loaded NPs, while non-coated counterparts were only effective after 48 h of treatment. The results were confirmed by histological analysis, highlighting that animals treated with drug-loaded NPs did not exhibit signs of both infection and inflammation when compared with non-treated infected animals. Despite these positive outcomes, the use of an immunosuppressed model restricts the conclusions regarding the role of the immune response on the treatment outcome or even any potential impact of NPs.

Souza et al. [67] proposed PLGA nanofibers (cross-section diameter of 638 nm) as vehicles for amphotericin B for controlling drug release under conditions simulating the vaginal environment. The use of nanofiber mats may be particularly relevant for vaginal drug delivery due to their similar use as, for example, vagina films used as contraceptives [85,86]. In the case of amphotericin B-loaded PLGA nanofibers, the antifungal was gradually released without the onset of a burst effect [67]. Such behavior was linked with the ability to achieve lower minimum inhibitory concentration (MIC) and minimum fungicidal concentration (MFC) values in vitro against *Candida* spp. as compared to the free drug. Nanofiber mats were tested in immunosuppressed (cyclophosphamide pre-treatment), pseudoestrus-induced Wistar rats previously inoculated intravaginally with *C. albicans* isolated from a patient. Drug-loaded nanofibers exhibited a roughly 3-log decrease in fungal burden after as little as 24 h of treatment, while complete eradication was only achieved after 3 days. Histological analysis confirmed complete elimination of hyphae on vaginal tissues, although there was still some infiltration of immune cells. Drug-free fiber mats were shown not to be effective, but the lack of additional controls–namely the free drug suspension or its formulation into a standard dosage form–limits the reach of presented data.

In another work, Ci et al. [68] proposed a nanocarrier-free approach in which the drug itself was nanosized and further dispersed in a thermosensitive gel based on a mix of poloxamers P407 and P188. The particles of amphotericin B were produced by a process involving high-speed shearing, sonication and high-pressure homogenization, and featured an average size of 247 nm and zeta potential around −30 mV. The gel was formulated in order to display a sol–gel transition around body temperature, thus allowing better retention in the vagina and prolonged drug release after administration. When tested in pseudoestrus-induced ICR mice challenged intravaginally with *C. albicans* CMCC 98001, the amphotericin B nanosuspension-in-thermogel was apparently effective after five daily vaginal treatments using different doses (1.25, 2.5 and 5 mg/kg). The evaluation was only performed one day after the last administration using subjective visual inspection and histological analysis of the vagina for spotting inflammation signs, which limits the value of the reported conclusions.

Nystatin is another polyene antifungal that is widely used for the topical treatment of VVC, especially in patients with cases of recurrence and azole resistance. However, its therapeutic efficacy may be limited by poor aqueous solubility, low stability, short vaginal residence time, and reduced penetration into infected tissues [87]. These obstacles can compromise drug residence and require prolonged treatment regimens that are prone to fail. Therefore, the use of nanotechnology-based delivery systems may be a promising alternative to optimize the vaginal administration of nystatin. For example, Hady et al. [69] proposed lipid vesicle nanostructures (termed transfersomes) as vehicles for improving the performance of the drug. Transfersomes are composed of phospholipids forming a lipid bilayer and single-chain surfactants that provide high flexibility to the structure. Nystatin-loaded transfersomes (350 nm, −54 mV) were tested in vivo in an albino mouse model of *Candida* vaginitis. Animals were first treated with estradiol valerate to induce a pseudoestrus state, and *C. albicans* 3153 A was inoculated after 72 h. Although the exact treatment regimen was not described, the authors claim that the nystatin-loaded transfersomes resulted in improved elimination of *C. albicans* as compared to the treatment with the free drug. Additionally, histological analysis demonstrated the maintenance of the normal epithelial structure, with low amounts of immune cell infiltration.

### 4.3. Plant Extracts

Revisiting ethnopharmacological principles and practices has been a prolific strategy for developing new medicines for the local treatment of VVC [88,89]. Various plant extracts were demonstrated to possess significant anti-*Candida* activity with potential for managing VVC, but their formulation may be hindered by their poor physicochemical properties (e.g., volatility in the specific case of essential oils, poor aqueous solubility or stability) and/or inherent toxicity [90,91,92,93,94]. Thus, incorporation of plant extracts into nanotechnology-based systems may be an interesting approach for converting antifungal plant extracts into usable topical products [95].

A few plant extract-containing nanosystems, namely nanoemulsions, have been tested in animal models of *Candida* vaginitis for their performance. For instance, Srivastava et al. [70] elaborated a carbomer-based gel formulation starting from a nanoemulsion (178 nm, −32 mV) containing spearmint (*Mentha spicata* L. var. *viridis*) essential oil. The in vivo effect of an optimized formulation was compared to that of clotrimazole (10 mg/mL dissolved in ethanol), using post-parturient Swiss albino mice pre-treated with cyclophosphamide and estradiol valerate (for induction of pseudoestrus) and challenged with *C. albicans* ATCC 14053. Specifically, the authors wanted to evaluate the efficacy of the nanoemulsion gel in managing vaginitis associated with pregnancy. The formulation was capable of significantly decreasing CFU count in vaginal lavages collected after treatment (once daily for 2 days) but was not as effective as clotrimazole. However, the relevance of the model to pregnancy-associated VVC is questionable since induction of pseudoestrus abbreviates the maintenance of the high progesterone environment typical of anestrus [96].

Another nanoformulation containing clove and tea tree essential oils has been proposed by Alkhanjaf et al. [71]. Combining antifungal and analgesic properties to appease genital symptoms, the oils were used to produce a nanoemulsion (62 nm, −40 mV) that was further incorporated into a thermosensitive gel. Single oil and combination gels were tested in vivo by intravaginally administering one gram of each formulation to pseudoestrus-induced oophorectomized rats at 24 h after inoculation with *C. albicans* (undefined strain or clinical isolate). The reduction in vaginal fungal burden was more pronounced over 21 days following a single administration in animals treated with the combination nanoemulsion gel, as compared to those treated with single active formulations. Still, the significance of these results is limited by the absence of a control group comprising standard-of-care (e.g., treatment with topical azoles). Additionally, evaluation of potential amelioration of vaginal irritation was not assessed.

**Figure 4:**
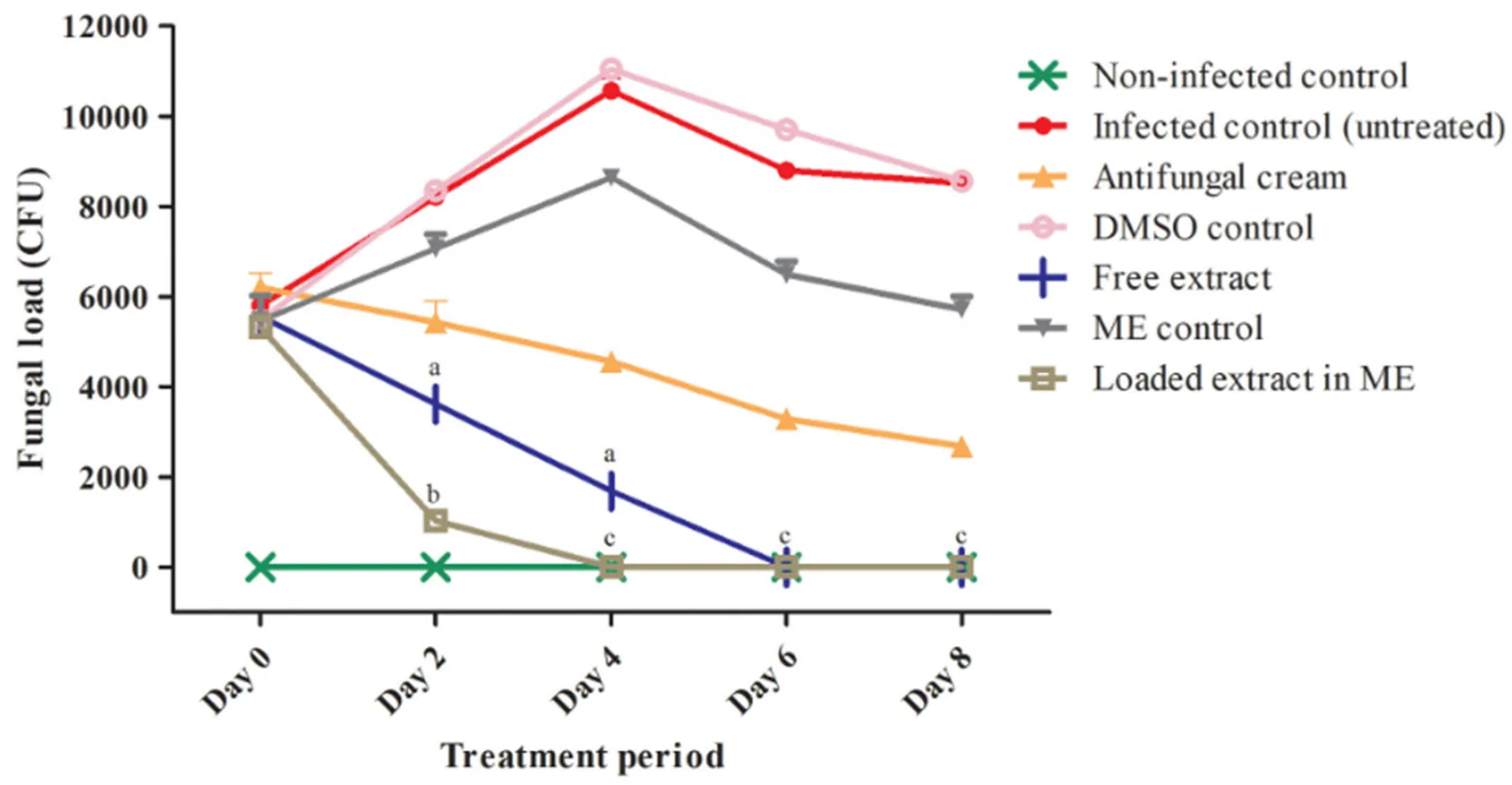
Fungal loads detected in vaginal lavages from animals in different experimental and control groups on days 0, 2, 4, 6 and 8 after treatment. Antifungal cream: contains tetracycline hydrochloride (25 mg/g) and amphotericin B (12.5 mg/g); DMSO: dimethyl sulfoxide; ME control: nanoemulsion without extract; loaded extract in ME: nanoemulsion with extract. Different letters in superscripts in columns indicate statistically significant differences (*p* < 0.05) according to two-way ANOVA with post hoc Tukey’s test. ^a^ Comparison between the free extract and the DMSO control (used as solvent for the free extract). ^b^ Comparison between the loaded extract and the nanoemulsion control. ^c^ Comparison between the loaded and the free extract. Adapted from [73], under the terms of the Creative Commons Attribution 4.0 International License (Copyright 2019, Bonifácio et al., https://doi.org/10.3389/fmicb.2019.02642).

Bonifácio et al. [72] proposed an optimized nanoemulsion (148 nm) containing a hydroethanolic extract of *Astronium urundeuva* leaves, which was shown to have considerable in vitro activity against azole-sensitive *C. albicans* ATCC 18804. The nanoemulsion was tested in an immunosuppressed (cyclophosphamide treatment), pseudoestrus-induced Wistar rat model of *Candida* vaginitis. A single-dose treatment was used (25 µg of extract in 0.1 mL of nanoemulsion), showing to be more effective than the plain extract (25 µg) or a cream containing tetracycline hydrochloride (2.5 mg) and amphotericin B (1.25 mg) at 4–6 days post-treatment. The authors did not evaluate the in vivo safety of the nanoemulsion, but in vitro data appear to support that the formulation could at least partially decrease the marked cytotoxicity of the native extract. In a follow-up study [73], the same research group tested a slightly modified formulation by including poloxamer 407 to enhance mucoadhesion. In vivo evaluation was conducted using the same animal model but infected with a *C. albicans* strain (ATCC SC5314) that is capable of forming robust biofilms. Twice-daily treatments with the nanoemulsion were performed for eight days. As in the previous study, the nanoemulsion was able to reduce intravaginal fungal burden more effectively (Figure 4). Furthermore—and although experiments regarding cytotoxicity and in vivo toxicity in an *Artemia salina* L. model (brine shrimp) were conducted—the safety of the nanoemulsion was not confirmed in mammals.

### 4.4. Inorganic Nanoparticles

Beyond the use of nanocarriers for antifungal drug delivery, other nanomaterials have been shown to be potentially interesting for managing VVC due to their intrinsic anti-*Candida* activity [97,98]. These include mostly metallic NPs but, despite many promising reports of in vitro activity, their testing in animal models of *Candida* vaginitis has been virtually nonexistent.

One notable exception has been the recent study by Yu et al. [74] investigating the in vivo effects of gold NPs (AuNPs) combined with fluconazole after vaginal administration to pseudoestrus-induced BALB/c mice challenged intravaginally with multidrug-resistant *C. albicans*. The authors utilized gold NPs (19 nm, +23 mV) generated from the reduction of chloroauric acid by *C. albicans* (Ca_AuNPs) and demonstrated their ability to increase the antifungal activity of different azoles, even against drug-resistant strains. These effects were correlated with higher intracellular accumulation of antifungal compounds when administered in combination with the membrane-disruptive Ca_AuNPs. Moreover, the nanomaterial possessed low cytotoxicity and hemolytic effects. Assessment of the in vivo efficacy of the combination of Ca_AuNPs and fluconazole was conducted following once-daily intravaginal administrations for three consecutive days. No information on the doses was provided. Still, the combination treatment resulted in lower fungal burden in vaginal lavages, as compared to mice treated with fluconazole or Ca_AuNPs alone, as well as a reduction in keratinization and inflammatory cell infiltration of mucosal tissues. Additionally, immunohistochemical analysis revealed a decrease in TNF-α levels with the combination treatment, thus supporting the possibility of the treatment reducing the inflammatory response. Overall, this study does not provide definitive proof of the usefulness of AuNPs alone for managing VVC but stands as a promising first approach for establishing intrinsically active nanomaterials as alternatives or adjuvants to topical antifungal drugs.

## 5. Concluding Remarks and Future Perspectives

Increasing global incidence and recurrence are causes of great concern for present and future clinical management of VVC. Despite all efforts made over the last decades for developing new antifungal drugs, progress has been meager and new approaches are urgently required. The use of nanotechnology is at the forefront for achieving such a purpose, but translation requires initial proof-of-concept at advanced preclinical stages, including in vivo demonstration of efficacy and safety. Despite the wide range of proposed nanosystems that can be found in the scientific literature, only a few have been tested in models of *Candida* vaginitis. Still, reported data seem to generally support the potential of drug-loaded nanocarriers to provide effective treatment options, while testing of nanomaterials possessing intrinsic activity against *Candida* spp. is virtually nonexistent. Either because of poor results (e.g., low efficacy, high toxicity) that are typically undisclosed publicly or simply by lack of experimental endeavors, it seems that such antifungal nanomaterials may be unable to show positive results in vivo.

Thinking ahead and taking into consideration the studies presented in this work, there seems to be a lack of fully established in vivo models of *Candida* vaginitis, particularly related to their true relevance to the infection observed in women. Indeed, most of the models do not seem to truly represent the pathophysiology of human VVC and, therefore, may be considered of restricted value [99,100,101]. Current models are able to sustain *Candida* colonization of the vagina, but not necessarily the typical traits of productive infection. At the same time, host immune response and other physiological features (e.g., pH) of rodent models differ substantially from the disease hallmarks in women. Therefore, it is imperative to continue developing more reliable animal models to expedite not only the development of nanotechnology-based therapies, but also of other medical approaches to manage VVC. Another aspect missing in the studies reviewed in this work relates to local and regional antifungal drug levels provided by nanocarriers. Guaranteeing that such compounds present sustained antifungal levels in vivo is paramount not only to achieve therapeutic efficacy and fungal eradication, but also to prevent the emergence of resistance [102].

Additionally, most of the studies reviewed in this work have not fully addressed safety and toxicity issues in vivo. This is a particularly important gap when considering prolonged antifungal therapy required for RVVC. While the natural onset of vulvovaginal inflammatory response in symptomatic episodes tends to abbreviate, to some extent, the concerns about host response to locally administered products, the issue gains another importance when suppressive therapy in the otherwise healthy genital tract is required. Indeed, lack of biocompatibility of commercial vaginal products used for various applications is a well-known problem that has been deserving increasing attention over recent years [103,104,105]. Safe formulation principles also need to be enforced early on in the development process of topical antifungal nanomedicines and biocompatibility should be demonstrated in animal models, namely in those infected by *Candida* species [106]. Data from rodents and non-human primates indicate that the vaginal administration of nanomaterials can be overall regarded as safe, with little to mild exposure beyond mucosal tissues [107,108,109,110,111,112,113]. Also, these studies suggest that direct distribution of nanomaterials to the upper genital tract seems limited but possible, including in pregnant mice. Indeed, a recent study by Irvin-Choy et al. indicates that PEG-PLGA NPs may reach and accumulate in the placenta and embryo when administered intravaginally to pregnant mice [114]. Thus, extensive studies are required to assess possible developmental and reproductive safety issues.

Finally, the translation question: how does the field build on available pre-clinical evidence and move towards clinical testing and use? While efficacy in animal models seems achievable, particularly for antifungal drug-loaded nanomedicines, several limitations and challenges remain. As discussed above, overall in vivo safety, pharmacokinetics and nanomaterial biodistribution testing is largely missing. Also, chemistry, manufacturing, and control (CMC) aspects need to be fully addressed on an individual basis, as well as regulatory filings and bioethics approval, before moving on to clinical trials. The use of nanomedicines does not seem to be, in itself, sufficient motive requiring changes in clinical protocol designs typically used for testing vaginal antifungal products. However, the innovative side of nanomedicines may create additional and paradoxical barriers to acceptance and risk perception by patients, clinicians, regulatory decision-makers and even pharmaceutical companies developing such products [115]. These barriers may be particularly hard to overcome when dealing with a disease such as VVC, for which a deep urgency for developing new topical treatment options is not established among major stakeholders.

## Figures and Tables

**Table 1:** Overview of nanosystems developed for managing VVC and tested in animal models of *Candida* vaginitis.

Nanosystems	Associated Drugs	Diameter ^1^	Zeta Potential ^1^	Animal Model (Estrous Cycle Control)	*Candida* spp.	Key Outcomes	Refs.
Chitosan-based NPs	Miconazole nitrate	207 ± 1 nm	+28.7 ± 0.5 mV	BALB/c mouse (estradiol valerate)	*C. albicans* ATCC 10231	Similar efficacy with 7-times lower drug amounts, as compared with a commercial miconazole nitrate cream	[62]
Chitosan-based NPs	Fluconazole and propolis	317 ± 15 nm	+37.4 ± 0.3 mV	BALB/c mouse (estradiol valerate)	*C. albicans* ATCC 10231	Similar efficacy to a commercial miconazole cream	[63]
Hyaluronic acid-coated polycaprolactone NPs	Miconazole	211 ± 3 nm	−53.2 ± 0.4 mV	Wistar rat (estradiol cypionate)	*C. albicans* ATCC 10231	Hyaluronic acid enhanced the antifungal effects of drug-loaded NPs	[64]
Poly(lactic-*co*-glycolic acid) NPs	Amphotericin B	252 ± 5 nm	−22.0 ± 0.8 mV	New Zealand rabbit (estradiol valerate)	*C. albicans* ATCC 10231	Combining NPs with ultrasound irradiation resulted in nearly complete *C. albicans* depletion	[65]
Hyaluronic acid-coated Eudragit RL100 NPs	Amphotericin B	148 ± 17 nm	−29.9 ± 3.8 mV	Wistar rat (estradiol cypionate)	*C. albicans* ATCC 14053	Coating with hyaluronic acid decreased the time to eradication of *C. albicans*, as compared to non-coated NPs	[66]
Poly(lactic-*co*-glycolic acid) nanofibers	Amphotericin B	638 ± 94 nm	N.R.	Wistar rat (estradiol cypionate)	*C. albicans* (clinical isolate)	Partial (60%) and complete fungal clearance after 6 h and 3 days following treatment, respectively	[67]
Nanosuspension (dispersed in thermosensitive gel)	Amphotericin B	247 ± 8 nm	−30 mV	ICR mouse (estradiol benzoate)	*C. albicans* CMCC 98001	Decreasing in inflammation achieved for the nanosuspension-in-thermosensitive gel	[68]
Transfersomes	Nystatin	350 ± 1 nm	−54.0 ± 7.6 mV	Albino mouse (estradiol valerate)	*C. albicans* 3153A	Tissue accumulation of nystatin was improved	[69]
Nanoemulsion (dispersed in hydrogel)	Spearmint essential oil	178 ± 1 nm	−31.6 ± 2.0 mV	Swiss albino mouse (estradiol valerate)	*C. albicans* ATCC 14053	Partial fungal clearance (76%), contrasting with full clearance by clotrimazole	[70]
Nanoemulsion (dispersed in thermosensitive gel)	Clove and tea tree oil	62 nm	−40.4 mV	Rat (oophorectomy + estradiol benzoate)	*C. albicans*	Combination of both oils promoted a decrease in vaginal fungal burden	[71]
Nanoemulsion	*Astronium urundeuva* leaves extract	147 ± 2 nm	N.R.	Wistar rat (estradiol)	*C. albicans* ATCC 18804	Led to significantly higher reduction in vaginal fungal burden as compared to amphotericin B	[72]
Nanoemulsion	*Astronium urundeuva* leaves extract	N.R.	N.R.	Wistar rat (estradiol)	*C. albicans* SC5314	Higher antifungal activity as compared to non-formulated extract	[73]
Gold NPs	N.A.	19 ± 2 nm	+23.4 ± 0.5 mV	BALB/c mouse (estradiol benzoate)	*C. albicans* (multidrug-resistant clinical isolate)	Nearly complete fungal elimination when used as an adjuvant to topical fluconazole	[74]

^1^ Data presented as mean ± standard deviation (when available); N.A.: not applicable; N.R.: not reported.

## Data Availability

No new data were created or analyzed in this study. Data sharing is not applicable to this article.

## Abbreviations

The following abbreviations are used in this manuscript:

AuNPs	Gold nanoparticles
Ca_AuNPs	*C. albicans*-generated gold nanoparticles
CFU	Colony-forming units
MFC	Minimum fungicidal concentration
MIC	Minimum inhibitory concentration
NAC	Non-*albicans Candida*
NPs	Nanoparticles
PBS	Phosphate-buffered saline
PEG	Polyethylene glycol
PLGA	Poly(lactic-*co*-glycolic)
RVVC	Recurrent vulvovaginal candidiasis
VVC	Vulvovaginal candidiasis
